# Activation of cytosolic RNA sensors by endogenous ligands: roles in disease pathogenesis

**DOI:** 10.3389/fimmu.2023.1092790

**Published:** 2023-05-24

**Authors:** Sarah Straub, Natalia G. Sampaio

**Affiliations:** ^1^ Centre for Innate Immunity and Infectious Diseases, Hudson Institute of Medical Research, Clayton, VIC, Australia; ^2^ Department of Molecular and Translational Sciences, School of Clinical Sciences, Monash University, Clayton, VIC, Australia

**Keywords:** RNA, interferon, MDA5, RIG-I, PKR, OAS (2′5′-oligoadenylate synthetase), ZBP1, LGP2

## Abstract

Early detection of infection is a central and critical component of our innate immune system. Mammalian cells have developed specialized receptors that detect RNA with unusual structures or of foreign origin – a hallmark of many virus infections. Activation of these receptors induces inflammatory responses and an antiviral state. However, it is increasingly appreciated that these RNA sensors can also be activated in the absence of infection, and that this ‘self-activation’ can be pathogenic and promote disease. Here, we review recent discoveries in sterile activation of the cytosolic innate immune receptors that bind RNA. We focus on new aspects of endogenous ligand recognition uncovered in these studies, and their roles in disease pathogenesis.

## Introduction

The ability of the immune system to discern ‘self’ from ‘non-self’ is critical for its effective function and protection from invading pathogens. The innate immune system utilizes specialized receptors, termed Pattern Recognition Receptors (PRRs), that detect ligands associated with pathogens and usually absent in the host, termed Pathogen Associated Molecular Patterns (PAMPs) (1).

Aberrant RNA species generated during viral infections constitute a group of PAMPs that is recognized by specific PRRs in the cytosol, which is the location of many replicating viruses (2). As viruses replicate, they can generate RNA species through non-canonical pathways (e.g. RNA-dependent RNA polymerases), and these RNAs may lack features that are present in host RNA, such as 5’ cap structures or specific methylation patterns. They may also generate double-stranded RNA structures, or other unusual structures normally absent from cells. Recognition of these RNA species by different receptors will trigger various immune and antiviral responses, including inflammatory cytokine release, cell death, and shutdown of cellular processes (3). The nature of the induced response will depend on the receptor being activated.

**Figure 1:**
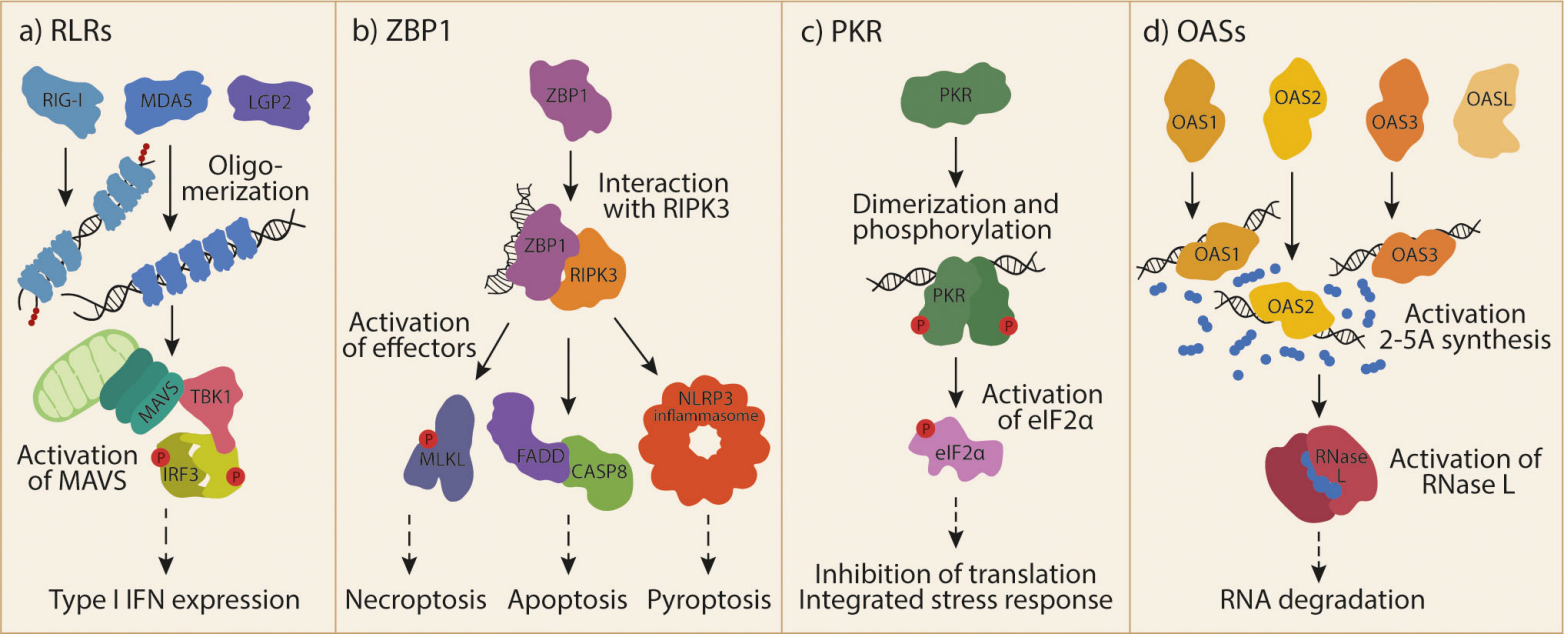
Cytosolic RNA receptor activation by dsRNA.**(A)** The family of RLRs includes RIG-I, MDA5 and LGP2. RIG-I and MDA5 can activate MAVS upon ligand recognition and oligomerization. Subsequently formed MAVS aggregates can activate TBK1 and IRF3 to induce T1-IFN responses. **(B)** ZBP1 is activated by Z-RNA and binds to RIPK3. This interaction allows the activation of different effector proteins and protein complexes, leading to different forms of cell death. **(C)** Recognition of endogenous dsRNA by PKR induces its dimerization and cross-phosphorylation. One of the most prominent targets of the activated kinase is eIF2α, whose phosphorylation leads to global inhibition of translation and the induction of the integrated stress response. **(D)** OAS1, OAS2, OAS3 make up the catalytically active part of the OAS family. OASL is a fourth, catalytically inactive member. Ligand recognition by OASs leads to their activation and synthesis of the second messenger 2-5A. 2-5A is bound by RNase L, inducing its dimerization and subsequent degradation of RNA. RIG-I, retinoic acid inducible gene I; RLRs, RIG-I-like receptors; MDA5, melanoma differentiation-associated gene 5; LGP2, laboratory of genetics and physiology 2; MAVS, mitochondrial antiviral-signaling protein; TBK1, TANK-binding kinase 1; IRF3, interferon regulatory factor 3; T1-IFN, type I interferon; ZBP1, Z-DNA-binding protein 1; RIPK3, receptor-interacting protein kinase 3; MLKL, mixed lineage kinase domain-like; NLRP3, NOD-, LRR- and pyrin domain-containing protein 3; FADD, FAS-associated death domain; CASP8, caspase 8; PKR, Protein kinase RNA-activated, dsRNA, double-stranded RNA; eIF2α, eukaryotic translation initiation factor 2A; OAS, 2’-5’-oligoadenylate synthetase; OASL, OAS-like; 2-5A, 2’-5’-oligoadenylate; RNAse L, ribonuclease L.

The cytosolic RNA receptors of the innate immune system can sense different types of RNA and induce different types of responses (**Figure 1**). The receptors known to date and discussed in this review are: 1) the family of RIG-I-like receptors (RLRs); 2) Z-DNA-binding protein 1 (ZBP1); 3) protein kinase RNA-activated (PKR); and 4) the family of 2’-5’-oligoadenylate synthetases (OASs). In addition to the cytosolic receptors, RNA can be sensed by other PRRs such as the transmembrane Toll-like receptors. These differ from the cytosolic receptors in their subcellular localization (e.g. in endosomes) and downstream signaling pathways, and will not be discussed in this review. The importance of the cytosolic RNA receptors in viral infection is well established (4). However, their involvement in non-infectious diseases is being increasingly recognized. How these receptors become activated in the absence of infection, what the likely sources of their endogenous ligands are, and what their roles in disease are will be discussed here.

## RNA sensing in non-infectious disease/sterile inflammation

Although RNA sensing is important in defence against pathogens, dysregulation or inappropriate activation of RNA sensing can also lead to disease. A well-known example is the group of diseases termed “type I interferonopathies”, which develop as a result of excessive and chronic production of type I interferons (T1-IFNs) (5). T1-IFNs are a group of cytokines that act on most cells in the body to induce the expression of hundreds of interferon stimulated genes (ISGs) and promote an antiviral state. Acute expression of T1-IFNs is important in protection from infection, but chronic production can cause pathogenic inflammation (6). Aicardi-Goutières Syndrome (AGS) was the first interferonopathy to be discovered, and can arise from mutations in different components of nucleic acid metabolism and sensing pathways, leading to uncontrolled pathogenic T1-IFN production (7). AGS is a severe neurological disease with variable features, including intracranial calcification and vasculitic skin lesions. Gain-of-function mutations in MDA5, for example, can cause AGS and other interferonopathies (8, 9). In addition to monogenic interferonopathies, other inflammatory or autoimmune diseases have also been associated with nucleic acid sensors, such as type 1 diabetes (10, 11) and inflammatory bowel disease (12, 13). Furthermore, their potential role in cancer is being increasingly recognized (10, 14). A clear lesson we can gleam from interferonopathies is that nucleic acid sensors, including RNA sensors, can be activated in the absence of infection (5). Importantly, this indicates that endogenous host RNAs can serve as ligands for these sensors, breaching the expected discrimination of self vs non-self in innate immune tolerance.

## Rig-I-like receptors

The RLR family of cytosolic RNA receptors constitutes retinoic acid inducible gene I (RIG-I; encoded by *DDX58*), melanoma differentiation-associated gene 5 (MDA5; encoded by *IFIH1*) and laboratory of genetics and physiology 2 (LGP2; encoded by *DHX58*). RLRs play an important role in protection from viral infection (4). RLRs detect double-stranded RNA (dsRNA), but with distinct molecular specificities. These receptors share a DExD/H-box RNA helicase domain and a C terminal domain (CTD). Additionally, RIG-I and MDA5 contain two N terminal caspase activation and recruitment domains (CARDs). Activation of RIG-I and MDA5 by an RNA ligand leads to homotypic interaction of their CARD domains with a CARD domain on the adaptor molecule mitochondrial antiviral-signaling protein (MAVS). Activated MAVS in turn oligomerizes and triggers a downstream signaling cascade involving TBK1, to activate interferon regulatory factors (IRFs) and the NF-κB pathway (4). This culminates in the expression of T1-IFNs, other cytokines, chemokines and antiviral factors. Release of T1-IFNs from the cell induces a potent pro-inflammatory and antiviral gene signature through autocrine and paracrine activation of their cognate cell surface receptors. LGP2 lacks CARD domains and therefore cannot activate MAVS, but interacts with MDA5 and RIG-I to promote and regulate their functions.

### MDA5

The RLR that has been most strongly implicated in sensing of host-derived RNAs is MDA5. Gain-of-function mutations in MDA5 cause a range of interferonopathies, including AGS (8, 9) as well as Singleton-Merten syndrome (SMS), which can include dental dysplasia, psoriasis, glaucoma, aortic calcification, and skeletal abnormalities (15). Recently, an expansive report of patients presenting with gain-of-function MDA5 mutations has highlighted the variability in disease expression and penetrance (16).

A requirement of MDA5 signaling is the oligomerisation of MDA5 monomers on a dsRNA molecule to allow cooperative signaling through the CARD domains (17). Biochemical studies of MDA5 bearing mutations found in AGS patients revealed that mutant MDA5 has a lower threshold for activation (18), and is able to promote oligomerisation with lower affinity ligands (19). It was shown using recombinant MDA5 protein in an *in vitro* approach, that this mutant MDA5 binds to host repetitive *Alu* retroelements that can occur in paired inverted orientation (IR-*Alus*). These IR-*Alus* can form dsRNA structures due to sequence complementarity, and stimulate MDA5. Indeed, repetitive RNA structures have been implicated in MDA5 sensing in other scenarios: treatment of cancer cells with DNA demethylating drugs induced the expression of endogenous retroviral elements (EREs), which also have the propensity to form dsRNA structures and thus activate MDA5 (20, 21). Recently, treatment of breast cancer cells using spliceosome-targeting therapies, which inhibit appropriate RNA splicing and promote intron retention in mRNA, was found to induce MAVS-dependent T1-IFN responses and apoptosis (22). These intron-containing mis-spliced RNAs have a tendency to form dsRNA structures, which activate dsRNA sensors, including MDA5. Another recent report demonstrated that genetic ablation of hnRNPC, a critical splicing regulator, also induced MDA5-dependent activation via increased expression of intronic RNA (23). This stimulatory intron-containing RNA was found to be enriched in *Alu* repeats. Together, these biochemical studies using mutant MDA5 and therapeutic interventions in cancer, demonstrate that repetitive elements are likely to be an important source of endogenous dsRNA that can be sensed by MDA5 in certain contexts.

Mitochondria, being an ancestral structure derived from symbiotic bacteria, can generate dsRNA species due to bidirectional transcription of its circular genome. An accumulation of mitochondrial dsRNA (mt-dsRNA) was found in the absence of mitochondrial RNA helicase SUV3 and polynucleotide phosphorylase PNPase, which mediate mt-dsRNA degradation (24). The mt-dsRNA activated MDA5 to trigger a T1-IFN response. Indeed, patients harbouring mutations in *PNPT1*, which encodes PNPase, have a T1-IFN signature, though it is not clear if this contributes to disease in these patients (25). Nevertheless, this work highlighted mt-dsRNA as a novel endogenous ligand for MDA5.

SAM domain and HD domain-containing protein 1 (SAMHD1) is a phosphohydrolase degrading deoxyribonucleoside triphosphate (dNTP), and loss of functional SAMHD1 can also cause AGS (26). Surprisingly, it has now been reported that SAMHD1 also functions as a single-stranded RNA (ssRNA) 3′exonuclease that is required to keep homeostatic RNA levels within the cell (27). Loss of SAMHD1 led to an accumulation of ssRNA within the cell, which dissolved the formation of RNA-protein condensates that sequester endogenous dsRNA. Release of dsRNA from these condensates then activated a T1-IFN response that was both MDA5 and RIG-I dependent. However, the origin or identity of the dsRNA causing this activation was not investigated.

The propensity of MDA5 activation by endogenous RNAs is best highlighted through the role of adenosine deaminase acting on RNA 1 (ADAR1). ADAR1 is an enzyme that binds dsRNA and catalyses the conversion of adenosine base to inosine (A-to-I editing). Inosine and uridine do not form hydrogen bonds, thus A-to-I editing reduces base pairing of dsRNA. ADAR1 knockout mice are embryonically lethal due to chronic production of T1-IFN, but can be rescued with concomitant knockout of MDA5 (28). ADAR1 has two isoforms: p110 is constitutively expressed and found in the nucleus, and the longer p150 is T1-IFN-inducible and primarily located in the cytoplasm (29). It is the p150 isoform that seems to protect against MDA5-mediated activation, as ADAR1-p150 knockout mice, like the full knockout, are embryonic lethal and rescued by loss of MDA5 (30). This seminal work established an ADAR1-dsRNA-MDA5 axis where ADAR1 functions to reduce base-pairing of endogenous dsRNA in order to prevent non-specific stimulation of MDA5. Furthermore, loss-of-function mutations in ADAR1 can also induce AGS, reinforcing this ADAR1-dsRNA-MDA5 axis (31).

Two recent works from the group of Jin Billy Li have shed light on the identity of endogenous MDA5-stimulating dsRNA in the context of ADAR1, and their broader role in common inflammatory and autoimmune diseases. Li et al. employed quantitative trait locus (QTL) mapping to define RNA-editing QTLs (edQTLs), thus linking RNA editing levels to disease traits (32). They detected colocalization of edQTLs and genetic variants associated with autoimmune and inflammatory conditions, such as inflammatory bowel disease, multiple sclerosis, and lupus. Importantly, linking of edQTLs to genome wide association studies (GWAS) of autoimmune and immune-related diseases showed that risk GWAS variants were associated with reduced dsRNA editing in the risk variant region. This suggests that an increase in unedited dsRNA can lead to greater risk of disease, presumably through induction of MDA5-dependent T1-IFN responses. The authors further characterized cis-natural antisense transcripts (cis-NATs) as a novel endogenous dsRNA. These are formed by bidirectional transcription at a genomic locus, which can lead to perfect base-pairing between the forward and reverse RNA strands. Cis-NATs were potent MDA5 agonists *in vitro*, and most were found to be hyper-edited in sequencing data, indicating their immunogenic potential and targeting by ADAR1. The authors also suggest that cis-NATs might be an important and preferred endogenous ligand for MDA5, in addition to IR-*Alus* previously linked to MDA5 mutants in AGS (18).

Additionally, a pre-print by Sun et al. experimentally investigated ADAR1-editing of endogenous dsRNA on a panel of genetically manipulated cells that lacked different ADAR1 isoforms and/or MDA5 (33). Using RNA-seq, they found that only a small proportion (1-2%) of dsRNA, which were normally A-to-I edited, were driving an immunogenic T1-IFN phenotype when unedited. The majority of these termed ‘immunogenic dsRNA’ were IR-*Alus*, but in contrast to previous work (18), were mostly in untranslated regions (UTR) or intergenic regions, rather than introns, consistent with a cytosolic localization. Immunogenic IR-*Alus* tended to have shorter sequences between the inverted *Alu* repeats, which presumably increase their base-pairing stability. The authors also confirmed that cis-NATs were very rare but highly immunogenic dsRNAs, due to their perfect base-pairing that induces strong activation of MDA5. Finally, the authors employed different human cells (HEK293T, neuronal progenitor cells, and hepatocyte-like cells), and human vs mouse ADAR1p150 overexpression systems, to investigate inter-cell and inter-species differences in dsRNA expression and editing. They demonstrated that differences in expression patterns of immunogenic dsRNAs across cell types and species drove the effects seen in stimulation of the ADAR1-dsRNA-MDA5 axis. Simply put, an immunogenic dsRNA will drive MDA5 activation when it is expressed in a particular cell, and this expression timing and level, in conjunction with ADAR1 activity and MDA5 presence, will drive a T1-IFN response. These recent advancements further define endogenous RNAs that activate MDA5 in the absence of virus infection, as well as link RNA sensing to specific disease states.

In addition to the dsRNA binding region of ADAR1, present in both p110 and p150 isoforms, the cytoplasmic p150 isoform also contains a Z-nucleic acid binding domain. The Z-form is a left-handed helix conformation of double stranded nucleic acids that is thermodynamically less stable compared to the more common right-handed helices in B-DNA and A-RNA structures in most conditions (34). Backbones of Z-helices form zig-zag structures and upside-down hydrogen bonding between strands due to changed base conformations, inspiring the structure’s name (35). Z-RNA forming sequences are often found in repetitive regions of the genome that, if transcribed, can form dsRNA structures that activate many of the nucleic acid sensors discussed in this article (36–39). Additionally, Z-nucleic acid formation is favoured by polymerase, helicase or topoisomerase activity (40) and interactions with nucleic acid binding proteins, such as FBP, FIR, BRG1, ADAR1 and potentially MDA5 (37, 41–43).

Three separate reports have demonstrated that the Z-RNA binding domain of ADAR1 contributes to its role in protecting cells from non-specific activation of MDA5. Using mouse models, these groups showed that loss of functional Z-RNA binding by ADAR1 led to MDA5/MAVS-dependent spontaneous T1-IFN induction (44–46). Differences in the *in vivo* models used showed discrepancies in the phenotypes detected. de Reuver et al. (44) and Tang et al. (46) used a mouse containing two point mutations (N175A and N179A) in the Zα domain of ADAR1, abrogating its Z-RNA binding capacity. This mouse was developmentally normal and born at expected mendelian ratios. In contrast, Nakahama et al. (45) introduced a single point mutation (W179A), which also abrogated Z-RNA binding, but in this case caused abnormal development and an AGS-like phenotype in the mice. Despite these differences, all groups reported that loss of Z-RNA binding affected A-to-I editing in some form, though this was in a minor proportion of sequences and they were challenging to categorize. In particular, de Reuver et al. reported that loss of editing was more pronounced in 3’ UTR regions as opposed to intronic regions. This supports the hypothesis put forward by Sun et al. (33) that the critical immunogenic dsRNAs edited by ADAR1 are those located in the cytoplasm. Collectively, these works indicate that Z-RNA binding by ADAR1 contributes to its ability to protect cells from deleterious MDA5-mediated T1-IFN production. However, it remains unclear whether Z-RNAs form a distinct subset of dsRNA species detected by ADAR1, or if Z-RNA binding supports full function of ADAR1 in detecting dsRNA in general.

### RIG-I

As the prototypic receptor of the RLR family, activation of RIG-I by viral infection has been widely investigated, and its RNA ligand is well defined, unlike that of MDA5 (4, 47). RNAs forming intra-molecular base-pairs, containing an uncapped triphosphate or diphosphate group at the 5’ end, and lacking 2’-O methylation at this site, are potent ligands of RIG-I. These RNA features are absent in host RNA, which is 5’ capped and/or 2’-O cap methylated. However, during some virus infection, with influenza A virus (IAV) being the best studied example, production of viral RNA by the viral RNA polymerase generates RNA species with these distinct features. These RNAs become potent RIG-I ligands.

Interestingly, it has been shown that DNA viruses, which are not expected to generate RIG-I stimulatory RNA due to lack of RNA-dependent RNA polymerase activity, could still stimulate RIG-I. Recent work has shed light on how this might occur. Infection of cells with herpes simplex virus 1 (HSV-1) or Epstein-Barr virus (EBV), both DNA viruses of the *Herpesviridae* family, activated RIG-I. It was found that this depended on cytoplasmic re-localization and release of nuclear host 5S ribosomal RNA pseudogene transcripts, induced via viral infection (48). Host 5S ribosomal RNA, transcribed by RNA polymerase III, contains 5’-triphosphates but is normally shieled from RIG-I by containment in the nucleus and by binding to specific proteins. This shielding was lost during infection, leading to RIG-I activation. It was also shown that infection with Kaposi’s sarcoma-associated herpesvirus (KSHV), another *Herpesviridae* family member, stimulated RIG-I. This occurred through virus-induced concurrent downregulation of the cellular triphosphatase DUSP11 and upregulation of noncoding vault RNAs (49). Vault RNAs are part of a cytoplasmic ribonucleoprotein complex called “vault”, who’s function remains enigmatic (50). In the absence of DUSP11, which removes 5’ triphosphate moieties, these vault RNAs retain the 5’-triphosphates that activate RIG-I. Similarly, host small non-coding Y RNAs were found to stimulate RIG-I during infection with the RNA viruses measles and dengue and the retrovirus human immunodeficiency virus 1 (HIV-1) (51). Y RNAs form stem loop structures, and are involved in DNA replication, RNA stability and cellular stress responses (52). This was also mediated by virus-induced downregulation of DUSP11, leading to an increase in 5’-triphosphate-containing host RNAs. Finally, it was shown that IAV infection decreased SUMOylation of TRIM28, a transcriptional regulator, which led to expression of EREs that induced a RIG-I-MAVS-mediated T1-IFN response (53). However, this activation only occurred in the absence of the IAV protein NS1, a major viral IFN antagonist known to inhibit RIG-I activation and signalling pathway (54). Combined, these studies demonstrate certain endogenous host RNAs are capable of stimulating RIG-I, albeit in the context of viral infection.

Although the interferonopathy SMS is more commonly associated with gain-of-function mutations in MDA5, activating mutations in RIG-I have also been found to cause SMS (55–57). These cases demonstrate that, like MDA5, RIG-I may be capable of sensing endogenous host RNA in the absence of infection. However, the requirement for specific molecular moieties on RNA, like the presence of 5’-triphosphates or absence of 2’-O methylated caps, makes it difficult to envision how RIG-I is activated in SMS. Recent biochemical approaches have revealed that SMS-causing mutations impair the ability of RIG-I to discern 2’-O methylated caps, lifting some of the checkpoints required for RIG-I activation (58, 59). This likely decreases the threshold for RIG-I activation, rendering it more susceptible to stimulation by endogenous RNAs. Nevertheless, the precise origin and nature of these endogenous RNA ligands remains unknown.

### LGP2

The least understood and studied receptor of the RLR family is LGP2. It can bind with different affinities to dsRNA of various lengths and structural features (60), but since it lacks an N-terminal CARD domain, it is unable to activate MAVS signaling and directly induce antiviral and inflammatory responses. Instead, it can interact with and alter the functions of RIG-I and MDA5 in opposing ways, suppressing the former while potentiating the latter (4, 61). Interestingly, binding of dsRNA is not required for LGP2s suppressive effect on RIG-I (60) and it could instead be determined through interactions with other RNA binding proteins. Further biochemical studies indicate that LPG2 can facilitate MDA5 filament formation on dsRNA, suggesting it plays a role in MDA5 ligand detection (62).

Similar to RIG-I, LGP2 has mostly been studied in the context of viral infection. However, recently it was revealed that LGP2 also plays a role in response to endogenous ligands in the context of ADAR1 loss and cancer therapy. Using *in vitro* cell models, Stok et al. (63) demonstrated that T1-IFN induction consequent of ADAR1 loss was equally dependent on LGP2 as it was on MDA5. Interestingly, the dependence on LGP2 for T1-IFN responses was limited to self-RNA ligands, as stimulation with the dsRNA mimic high molecular weight (HMW) poly(I:C) or with RNA isolated from EMCV-infected cells partially bypassed the requirement of LGP2 for MDA5 activation. The authors hypothesize that LGP2 may be required for T1-IFN responses to the ‘weaker’ self-RNA ligands, but dispensable in the sensing of ‘stronger’ ligands, such as those generated by viral infection. They further demonstrated that LGP2 was essential for the T1-IFN response in cancer cells induced by concomitant ADAR1 loss and treatment with DNA methyltransferase inhibitors, the latter previously shown to induce MDA5 activation through sensing of de-repressed EREs (20, 21). Thus, through its cooperative role in MDA5 activation, LGP2 may indeed be involved in sensing of self-RNA ligands. These endogenous ligands are likely to be the same as those sensed by MDA5, though this has yet to be formally demonstrated.

## ZBP1

ZBP1 is a specialized nucleic acid sensor that recognizes and binds double-stranded nucleic acids in Z-conformation via two N-terminal Zα-domains (64, 65). Initial research investigated the DNA-binding activity of ZBP1, but its more prevalent role as an RNA sensor has been the main focus of recent studies (66). Activation of ZBP1 during infection induces innate immune or inflammatory responses as well as different forms of cell death. ZBP1 activation facilitates interactions with receptor-interacting protein kinase (RIPK) 1 and RIPK3 via two RHIM domains. This can lead to mixed lineage kinase domain-like (MLKL) activation and necroptosis, FAS-associated death domain (FADD)-caspase 8-mediated apoptosis and NOD-, LRR- and pyrin domain-containing protein 3 (NLRP3) inflammasome-mediated pyroptosis (67–71).

More recently it has been shown that ZBP1 can be activated by endogenous dsRNA in the context of infection, but also in sterile settings (12, 68, 72, 73). Using immortalized MEFs, Jiao and colleagues (72) showed that the Zα domains of ZBP1 bind dsRNA, most likely derived from EREs. The Zα domains also induced necroptosis and contributed to the inflammatory phenotype of FADD^IEC-KO^ colitis mice and Ripk1^E-KO^ skin inflammation mice. They further showed that caspase-8 can be recruited to the to the ZBP1-RIPK3 complex via FADD and RIPK1. Caspase-8 can then prevent activation of ZBP1-RIPK3 and inhibit necroptosis (72). Another study confirmed the contribution of ZBP1-induced necroptosis in response to sensing of endogenous dsRNA in the Ripk1^E-KO^ mouse skin inflammation model, and also discovered a role for type 1 and type 2 IFNs (73). High levels of ISGs, including ZBP1, were detected in skin lesions, and phosphorylation of RIPK3 and MLKL was induced by IFNs. A similar observation was made in a study investigating the role of the methyltransferase SETDB1, a repressor or EREs, in IBD (12). A reduction of SETDB1 levels in intestinal stem cells in mice led to increased expression and accumulation of EREs. EREs can form double-stranded Z-RNA segments that activate ZBP1 and subsequent RIPK3-dependent necroptosis, which promoted bowel inflammation by damaging the epithelial barrier (12).

The accumulation of EREs in the cytoplasm is also observed in response to loss of ADAR1 in mice, such as Adar1^mZa/-^ mice (74). Adar1^mZa/-^ mice carry only one ADAR1 allele with a mutated Zα domain, and ZBP1 and MAVS have been shown to contribute to its postnatal lethality. Loss of ADAR1 led to an increased expression of ISGs and an accumulation of EREs, which activated RLRs and ZBP1, leading to excessive inflammation (74). A similar increase in ISG expression in different organs has been observed in Adar1^mZa/mZa^ mice and was shown to be dependent on MAVS (46). The additional deletion of ZBP1 in Adar1^mZa/-^ mice prevented ISG expression and ERE accumulation, an effect that could interestingly only be partially mimicked through deletion of RIPK3 and partial deletion of FADD, suggesting the involvement of a different downstream mechanism than the ones described previously (74).

Different ADAR1 mutations result in a similar phenotype, such as the ones seen in Adar1^P159A/p150null^ mice. Perinatal lethality of this genotype is dependent on activation of both MDA5 and ZBP1, resulting in RIPK3-dependent and independent cell death (75). Another study confirmed the involvement of both MAVS and ZBP1 in the development of the severe ADAR1 knockout autoinflammatory phenotype in mice, as well as the inhibitory role of the ADAR1 Zα domain on ZBP1 activation (44). In bone marrow derived macrophages, ADAR1 further regulated ZBP1-activation by endogenous ligands by competing with RIPK3 for ZBP1 binding, which limits PANoptosis (76). A direct interaction of ADAR1 with ectopically expressed ZBP1 has subsequently also been shown in HEK293T cells and was dependent on intact ZBP1 Zα-domains (75).

In addition to its role in auto-inflammatory conditions, ZBP1 also plays a role in the suppression of tumorigenesis through treatment with IFNγ and nuclear export inhibitors in mice, or in the absence of ADAR1 (76). This activity is dependent on ZBP1’s dsRNA-binding domain Zα2 and is therefore likely dependent on the recognition of endogenous ligands. Furthermore, ZBP1 activity has recently been found to be a crucial component in immune checkpoint blockade-based (ICB) therapy (77). The discovery was linked to ADAR1 deficiency, which led to the accumulation of 3’-UTR-derived EREs. These EREs can fold into double-stranded Z-RNA forming dumbbell-like structures and activate ZBP1, leading to RIPK3-dependent necroptosis. Since current ADAR1 inhibitors are not clinically useable, the authors investigated CBL0137, a small molecule activator of ZBP1. They showed that treatment with the activator induced ZBP1-activating Z-DNA formation and reversed resistance to ICB therapy in ADAR1 deficient cells (77). Similarly, a study investigating the natural flavonoid fisetin showed that its growth inhibitory effect on ovarian cancer cells, and expression of RIPK2 and MLKL, were mediated by ZBP1 (78).

Taken together, these recent findings on recognition of endogenous ligands by ZBP1 jumpstarted investigations on its contribution in sterile settings, such as tumorigenesis, as well as an array of inflammatory conditions. Understanding in more detail the role of ZBP1 in these disease settings will enable future clinical studies, and the development or improvement of treatments (77, 78). It is particularly interesting to consider how self-RNA ligands such as EREs might be activating ZBP1 in different tissue and disease contexts instead of, or in addition to, other PRRs like MDA5, given that activation of these receptors induces different downstream signaling events.

## PKR

PKR (encoded by *EIF2AK*) is an IFN-inducible, antiviral dsRNA-sensing kinase that is constitutively expressed at a low level (79). PKR generally binds to dsRNA longer than 30 base pairs (bp) and subsequently undergoes conformational changes that allow dimerization. PKR dimers are activated through auto-phosphorylation or phosphorylation by a neighbouring PKR dimer bound to the same dsRNA molecule; the dimer conformation does not allow *trans*-phosphorylation (80). PKR can be activated in response to viral infection, but also in uninfected cells undergoing mitosis when it comes into contact with IR-*Alus* upon disruption of the nuclear structure (81).

Activated PKR phosphorylates eIF2α to suppresses global translation and induce the integrated stress response (ISR) through the activation of transcription factors, like activating transcription factor 4 (ATF4), which enable return to cellular homeostasis or, under sustained stress, induce cell death (82, 83). PKR can also phosphorylate different mitotic factors, Histone H3 and c-Jun N-terminal kinase, which are crucial factors for cell cycle progression (81).

Endogenous nuclear-derived dsRNA can accumulate and activate PKR and its downstream targets in response to different stressors and conditions. Two recently published examples are ADAR1 dysfunction and exposure to phosphorothioate-modified antisense oligos (ASOs), which induce PKR-dependent eIF2α phosphorylation (84–86). The postnatally lethal phenotype and ISR and ISG expression signature of Adar1^P195A/p150-^ mice, which lack the catalytically active, IFN-inducible Adar1 isoform p150, has previously been described as MDA5-dependent (30). A recent publication by Maurano et al. (86) showed that LGP2, IFNAR1 and PKR are also essential factors mediating postnatal lethality and induction of ISR genes in Adar1^P195A/p150-^ mice, while PKR was dispensable for ISG expression. Postnatal mortality in these mutant mice could be prevented by feeding an ISR inhibitor that prevented translational arrest via eIF2α. Similar PKR-dependent ISR transcript upregulation in response to IFNβ was seen in an analogous, CRISPR/Cas9-based A549 cell model and overall suggests PKR is a downstream effector of the MDA5 and LGP2-induced IFN response (86). This hypothesis is further supported by a separate study investigating key mediators of anti-tumour immunity in ADAR1-null B16 cells, which naturally secrete IFNβ and undergo growth arrest (85). A CRISPR/Cas9-based knock out screen revealed suppression of this phenotype by a few different IFN-signaling components and PKR. This was confirmed using double knockout ADAR1 and PKR null B16 cells, which rescued the growth arrest phenotype, but did not prevent IFNβ secretion. Depletion of MDA5, RIG-I and MAVS on the other hand did not reverse growth arrest but prevented IFNβ secretion (85). These findings highlight the potential crosstalk between PKR and MDA5 activation.

PKR can also be activated by mt-dsRNA, as previously described for MDA5. Cellular stress or mitochondrial damage can lead to the release of mt-dsRNA into the cytoplasm, which has been shown to activate MDA5 in some cases (24). In contrast, PKR is partially localized to the mitochondrial matrix and can be activated there upon binding of mt-dsRNA under normal conditions (87). The degree of PKR activation in this context is regulated by the cell cycle: it increases in M-phase compared to S-phase, which coincides with an increase in mitochondrial RNA (mtRNA). Interestingly, the majority of PKR-bound mt-dsRNA in this study is derived from intramolecular duplex structures, not complementary strands expressed from bidirectional transcription of mitochondrial DNA. The study further showed that retention of activated PKR in the mitochondria prevented downstream effector phosphorylation (87). This work thus suggested a role for PKR in sensing cellular homeostasis through binding of specific endogenous mtRNA that may be released in conditions of cellular stress.

In a subsequent study, it was reported that disruption of mitochondrial membrane potential and structure by small molecule inhibitors led to PKR-dependent ISG induction, eIF2α phosphorylation and cell death (88). Further investigation showed that the induction of ISGs was dependent on MAVS and STING as well as PKR expression, but only PKR mediated eIF2α phosphorylation and cell death. The authors also showed that disruption of the mitochondrial membrane potential and PKR-binding to mt-dsRNA was dependent on mitochondrial RNA-polymerase activity and the expression of the mitochondrial pore-forming protein BAK1. Senescent, cultured chondrocytes and osteoarthritis patient-derived chondrocytes responded similarly to disruption of membrane potential. They showed an increased release of mt-dsRNA, PKR and eIF2α phosphorylation, and PKR-dependent induction of IL8 and MMP13, two catabolic factors associated with the development and progression of primary osteoarthritis. These responses could be alleviated by pre-treatment with autophagy-inducing inhibitors to temporarily remove stimulatory mtRNA (88). These studies showed that activation of different PRRs can have different downstream effects, and that their activation by similar ligands is dependent on subcellular localization and trafficking of the receptor and ligands.

In a recent study, another class of duplex structure-forming RNAs, the circular RNA (circRNA) was described in context of PKR (89). circRNAs are products of mRNA splicing and are very stable compared to linear RNA that is targeted by most cellular RNases. PKR was found to recognize very short and imperfect 16-26 nucleotide dsRNA regions formed by circRNAs, which led to its inhibition. The authors show that circRNAs in HeLa cells could be degraded by RNase L in response to EMCV infection or transfection of poly(I:C). This led to a rapid decline in global circRNA levels, because their biogenesis rate is very slow. A decreased level of circRNAs led to spontaneous PKR activation and inflammation in SLE-patient derived cells, which could be prevented by circRNA overexpression, highlighting their potential role in the development of future SLE treatments (89).

Different dsRNA structures are not the only endogenous, activating ligands for PKR. PKR can be activated by direct interaction with other ligands in the absence of dsRNA, such as the protein activator of PKR (PACT). Cellular stress can induce phosphorylation of PACT which leads to increased interaction with and activation of PKR. This can result in osmotic stress induced expression of pro-inflammatory genes (90–92). A more recent discovery is PKR activation through sensing of misfolded IL-24, which accumulates in the cytoplasm in response to proteotoxic stress (93). The authors generated a model for proteasome-associated autoinflammatory syndromes (PRAAS) through knock out of the immunoproteasome subunits iβ1 and iβ5 in THP-1 cells. They noted a PKR-dependent, but MAVS and STING independent inflammatory profile and increased IFNβ secretion in their model. The investigation of IL24 knock out cells with proteasome inhibitors showed an IL24 dependency of eIF2α phosphorylation, which led to an inflammatory response (93). Further to this, misfolded IL24 escaped the ER and accumulated in the cytoplasm in response to proteasome inhibition. The mechanism behind misfolding has not yet been identified, but analyses of PRAAS patient samples revealed increased levels of IL24 that was associated with PKR following immunoprecipitation. This study shows that proteasome dysfunction is a key driver of inflammatory responses in PRAAS patients and that inhibition of PKR using small molecules could alleviate that response (93).

The recent findings on sterile PKR activation described here clearly show the large number and variety of endogenous ligands recognized by PKR. Many of these interactions are cell-type and context dependent and require further investigation and characterization to understand them in detail. However, the studies have already shown their future potential in treating PKR-mediated autoimmune and autoinflammatory diseases.

## OASs

The OASs are a family of IFN-inducible cytosolic dsRNA sensors consisting of the catalytically inactive OASL and the catalytically active OAS1, OAS2 and OAS3 proteins. OASL has antiviral functions, despite being catalytically inactive, which are mediated by interaction with and activation of RIG-I through mimicking polyubiquitin (94). OAS1, OAS2 and OAS3, however, undergo conformational changes and dimerization upon recognition of dsRNA and synthesize 2’5’-linked oligoadenylates (2-5A) in an ATP-dependent manner (95–97). 2-5A acts as a second messenger molecule that activate RNase L, a ribonuclease that dimerizes and indiscriminately degrades both viral and cellular single-stranded RNA, and induces apoptosis (98). Cleavage and decay of viral RNA limits virus replication, while degradation of cellular RNA limits translation and promotes expression of innate immune response genes (84, 99–101).

Research to date suggests the OAS isoforms have different affinities for different dsRNA, which is predominantly based on structure and length. OAS1 has been reported to have a binding preference for shorter dsRNA (≥17 bp), while OAS2 appears to require a minimum of 35 bp duplex structures and its target affinity increases with increasing dsRNA length (102). OAS3 on the other hand has a high affinity for RNA duplex structures longer than 50 bp, but its affinity for larger dsRNA sequences decreases, such as for a recently published 112 bp structure generated by *in vitro* transcription that resembles viral dsRNA (84, 97, 103, 104). These studies were largely conducted with recombinant proteins *in vitro*, thus precise delineation of OAS ligands in cells is still lacking.

Similar to the previously discussed dsRNA sensors, OAS overactivation or dysfunction is associated with autoinflammatory diseases, such as Alzheimer’s disease and AGS, as well as a higher risk of development of severe disease outcomes like COVID-19 (105, 106). Recent findings show activation of OAS family members by endogenous dsRNA in response to different stressors in the absence of viral infection (84, 105). Small molecule inhibitors of DNA methyltransferases induced hypomethylation of DNA and increased expression of EREs in both directions. Subsequently formed dsRNA accumulated in the cytoplasm, led to activation of caspase-3/7, PARP cleavage, and induced cell death within 48h (105). This phenotype was also shown in response to knock out of ADAR1 and the RLR signaling mediator MAVS in A549 cells. In both cases, induction of cell death was dependent on the catalytic activity of RNase L and the expression of at least one of the catalytically active OAS isoforms. Knocking out AKP7 or PDE12, 2-5A-targeting phosphodiesterases, or transfecting cells with 2-5A further promoted RNase L-dependent cell death, while inhibition of JNK prevented it (105). JNK can be activated by the pro-apoptotic kinase DRAK1, whose expression was induced by RNase L activation in response to transfection of A549 cells with poly(I:C) or 2-5A (107). This shows that OASs and RNase L can be activated by endogenous ligands of similar origin as those activating other PRRs discussed previously. Therefore, OAS activation may also contribute to the development of some autoinflammatory diseases, and could be a potential target for development of future treatments, though the link is not as well established as for other cytosolic RNA receptors.

A separate study described a potential role for OAS3 and RNase L in RNA homeostasis (84). The authors discovered PKR and OAS3-dependent RNase L activation in A549, HeLa and human lung epithelial cells after exposure to phosphorothioate (PS)-modified ASOs. This activation was driven by increased expression of intronic and intergenic endogenous RNA, termed endo-RNA. In particular, treatment of cells with PS-modified ASOs induced accumulation of promoter upstream region RNAs (PROMPTs) and RNA polymerase read-through transcripts. The authors were not able to demonstrate how PS-modified ASOs induced endo-RNA accumulation, but speculate that this could be through inhibition of RNA decay, nuclear retention and/or intron sorting, leading to their escape to the cytosol. RIG-I and MDA5 were not activated in this context, despite release of some of the dsRNA into the cytoplasm, suggesting that the accumulated endo-RNAs were specific OAS3/PKR ligands. A large proportion of the investigated sequences contained repeats that were predicted to form RNA duplex structures larger than 50bp, the preferred target of OAS3. In line with this prediction, RNase L was activated after transfection of a chemically synthesized RNA molecule containing a 65 bp inverted intronic repeat sequence. The accumulated RNA was also predicted to contain RNase L cleavage site in proximity to RNA duplex forming structures, making it both the predicted activator and target of the OAS3/RNase L axis (84). Importantly, the authors speculate that current FDA-approved ASOs might be inadvertently activating the PKR and OAS3-RNaseL pathway, as they demonstrated with the Vitravene, an antiviral ASO drug for patients with AIDS. Interestingly, changes to the expression of OAS1 and OAS2 did not contribute to RNase L activation in this study. This opens up the possibility of OAS isoform-specific sensing of endogenous dsRNA, an area that is also heavily investigated in the context of antiviral effects of OAS isoforms against different viruses (108–110). Defining the functional distinctions and overlaps of the OAS isoforms is an ongoing area of research, as is the nature and source of the dsRNA they recognize.

## Conclusions and open questions

**Figure 2:**
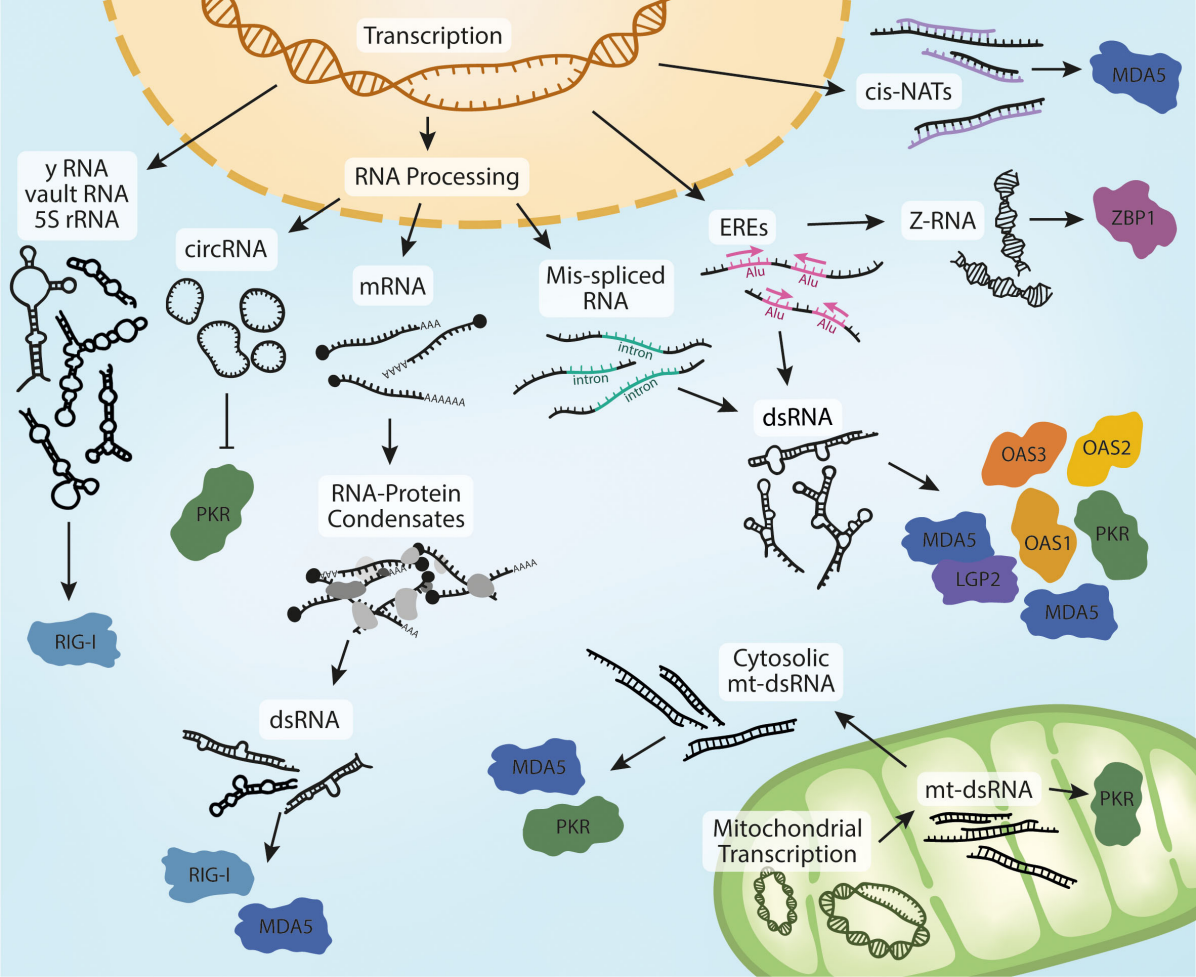
Biogenesis of endogenous RNA ligands. Nuclear transcription of Y RNA, vault RNA and the release of 5S rRNA into the cytoplasm in response to viral infection can activate RIG-I. Processing of pre-mRNA leads to the production of circRNA, potent ligands of PKR. Mature RNAs can accumulate in the cytoplasm and form RNA-protein condensates if their homeostasis is dysregulated. This can lead to the sequestration of MDA5- and RIG-I-activating dsRNA. Dysregulation of RNA processing is sometimes characterized by an increase in mis-spliced RNAs that retain introns. These RNAs can form intramolecular dsRNA structures and activate multiple receptors in different physiological contexts and models.The accumulation of dsRNA is also observed in response to increased transcription of EREs, which is often mediated by IR-Alu sequences. Increased ERE expression also leads to the formation dsRNA in Z-conformation, a ligand of ZBP1. The last reported nuclear derived species of MDA5-activating dsRNAs are cis-NATs, formed through bidirectional transcription and hybridization of complementary RNA strands. Further to these, MDA5 and PKR have also been reported to be activated by mt-dsRNA. Bidirectional expression of mitochondrial DNA leads to the formation of mt-dsRNA. mt-dsRNA can activate mitochondrial PKR, which is subsequently exported. It can also escape to the cytosol upon mitochondrial damage or oxidative stress, where it can activate cytosolic PKR and MDA5.

The recent wave of new discoveries on the activation of cytosolic RNA receptors in non-infectious disease has started to uncover the identity and structure of endogenous ligands of these receptors (**Figure 2**). It is noteworthy that many of these receptors overlap in both the source of endogenous ligands and in the sterile diseases they are implicated in. For example, rare monogenic interferonopathies (e.g. AGS) as well as some more common conditions (e.g. inflammatory bowel disease) have been linked to the activation of multiple receptors in. It is also interesting that endogenous RNAs such as EREs, IR-*Alus*, and mt-dsRNA have been reported as ligands for several receptors. Similarly, cancer treatments that generate endogenous RNA ligands have also revealed roles for different receptors. Since most of these phenomena have been investigated in isolation, it remains unclear whether these same ligands activate multiple receptors simultaneously, and how potential cooperative signaling from these receptors might affect disease outcome.

Structural and *in vitro* studies have been imperative to our understanding of how the cytosolic RNA receptors work. However, it is evident that receptor activation in sterile disease is context-dependent and requires *in situ* studies. Importantly, experiments using endogenous proteins and in disease-relevant tissues will be necessary to fully delineate the endogenous ligands of these receptors. The many technical and methodological advancements of the last few years should facilitate such investigations, such as SHAPE-MaP, a tool developed to determine sequence and structure of RNA ligands, or iCLIP, a method to investigate the precise interaction of RNA ligands with their receptors. Combining these different methods and applying them in disease-specific human tissues, for example iPSC-derived models or organoids, could reveal the exact ligands of each of these receptors in appropriate physiological contexts, which remains unknown in many cases.

Furthermore, several receptors are expressed in the same cell type and tissue and appear to share activating ligands, suggesting a potential for combined activation. Evaluating the contribution of the different receptors in these settings will be an important aspect of future studies. Such investigations will broaden our understanding of innate immunity in non-infectious contexts, and open opportunities for novel treatment strategies.

## Author contributions

NS and SS wrote and revised the manuscript. All authors contributed to the article and approved the submitted version.

## References

[1] Akira S Uematsu S Takeuchi O. Pathogen recognition and innate immunity. Cell (2006) 124(4):783–801. doi: 10.1016/j.cell.2006.02.015 16497588

[2] Goubau D Deddouche S Reis e Sousa C. Cytosolic sensing of viruses. Immunity (2013) 38(5):855–69. doi: 10.1016/j.immuni.2013.05.007 PMC7111113 23706667

[3] Hartmann G. Nucleic acid immunity. Adv Immunol (2017) 133:121–69. doi: 10.1016/bs.ai.2016.11.001 PMC7112058 28215278

[4] Rehwinkel J Gack MU. RIG-i-like receptors: their regulation and roles in RNA sensing. Nat Rev Immunol (2020) 20(9):537–51. doi: 10.1038/s41577-020-0288-3 PMC7094958 32203325

[5] Crow YJ Stetson DB. The type I interferonopathies: 10 years on. Nat Rev Immunol (2022) 22(8):471–83. doi: 10.1038/s41577-021-00633-9 PMC8527296 34671122

[6] McNab F Mayer-Barber K Sher A Wack A O'Garra A. Type I interferons in infectious disease. Nat Rev Immunol (2015) 15(2):87–103. doi: 10.1038/nri3787 25614319 PMC7162685

[7] Crow YJ Manel N. Aicardi-goutières syndrome and the type I interferonopathies. Nat Rev Immunol (2015) 15(7):429 – 40. doi: 10.1038/nri3850 26052098

[8] Rice GI Duany Y Jenkinson EM Forte GMA Anderson BH Ariaudo G. Gain-of-function mutations in IFIH1 cause a spectrum of human disease phenotypes associated with upregulated type I interferon signaling. Nat Genet (2014) 46(5):503–9. doi: 10.1038/ng.2933 PMC4004585 24686847

[9] Oda H Nakagawa K Abe J Awaya T Funabiki M Hijikata A. Aicardi-goutières syndrome is caused by IFIH1 mutations. Am J Hum Genet (2014) 95(1):121–5. doi: 10.1016/j.ajhg.2014.06.007 PMC4085581 24995871

[10] Smyth DJ Cooper JD Bailey R Field S Burren O Smink LJ. A genome-wide association study of nonsynonymous SNPs identifies a type 1 diabetes locus in the interferon-induced helicase (IFIH1) region. Nat Genet (2006) 38(6):617–9. doi: 10.1038/ng1800 16699517

[11] Nejentsev S Walker N Riches D Egholm M Todd JA. Rare variants of IFIH1, a gene implicated in antiviral responses, protect against type 1 diabetes. Science (2009) 324(5925):387–9. doi: 10.1126/science.1167728 PMC2707798 19264985

[12] Wang R Li H Wu J Cai Z-Y Li B Ni H. Gut stem cell necroptosis by genome instability triggers bowel inflammation. Nature (2020) 580(7803):386–90. doi: 10.1038/s41586-020-2127-x 32296174

[13] Huang H Fang M Jostins L Umićević Mirkov M Boucher G Anderson CA. Fine-mapping inflammatory bowel disease loci to single-variant resolution. Nature (2017) 547(7662):173–8. doi: 10.1038/nature22969 PMC5511510 28658209

[14] Nabet BY Qiu Y Shabason JE Wu TJ Yoon T Kim BC. Exosome RNA unshielding couples stromal activation to pattern recognition receptor signaling in cancer. Cell (2017) 170(2):352–66.e13. doi: 10.1016/j.cell.2017.06.031 28709002 PMC6611169

[15] Rutsch F MacDougall M Lu C Buers I Mamaeva O Nitschke Y. A specific IFIH1 gain-of-function mutation causes singleton-merten syndrome. Am J Hum Genet (2015) 96(2):275–82. doi: 10.1016/j.ajhg.2014.12.014 PMC4320263 25620204

[16] Rice GI Park S Gavazzi F Adang LA Ayuk LA Van Eyck L. Genetic and phenotypic spectrum associated with IFIH1 gain-of-function. Hum Mutat (2020) 41(4):837–49. doi: 10.1002/humu.23975 PMC7457149 31898846

[17] Wu B Peisley A Richards C Yao H Zeng X Lin C. Structural basis for dsRNA recognition, filament formation, and antiviral signal activation by MDA5. Cell (2013) 152(1-2):276–89. doi: 10.1016/j.cell.2012.11.048 23273991

[18] Ahmad S Mu X Yang F Greenwald E Park JW Jacob E. Breaching self-tolerance to alu duplex RNA underlies MDA5-mediated inflammation. Cell (2018) 172:797–810. doi: 10.1016/j.cell.2017.12.016 PMC5807104 29395326

[19] Yu Q AHd V Singh R Modis Y. MDA5 disease variant M854K prevents ATP-dependent structural discrimination of viral and cellular RNA. Nat Commun (2021) 12(1):6668. doi: 10.1038/s41467-021-27062-5 34795277 PMC8602431

[20] Chiappinelli KB Strissel PL Desrichard A Li H Henke C Akman B. Inhibiting DNA methylation causes an interferon response in cancer via dsRNA including endogenous retroviruses. Cell (2015) 162(5):974–86. doi: 10.1016/j.cell.2015.07.011 PMC4556003 26317466

[21] Roulois D Yau HL Singhania R Wang Y Danesh A Shen SY. DNA-Demethylating agents target colorectal cancer cells by inducing viral mimicry by endogenous transcripts. Cell (2015) 162(5):961–73. doi: 10.1016/j.cell.2015.07.056 PMC4843502 26317465

[22] Bowling EA Wang JH Gong F Wu W Neill NJ Kim IS. Spliceosome-targeted therapies trigger an antiviral immune response in triple-negative breast cancer. Cell (2021) 184(2):384–403.e21. doi: 10.1016/j.cell.2020.12.031 33450205 PMC8635244

[23] Herzner A-M Khan Z Nostrand ELV Chan S Cuellar T Chen R. ADAR and hnRNPC deficiency synergize in activating endogenous dsRNA-induced type I IFN responses. J Exp Med (2021) 218(9):e20201833. doi: 10.1084/jem.20201833 34297039 PMC8313407

[24] Dhir A Dhir S Borowski LS Jimenez L Teitell M Rötig A. Mitochondrial double-stranded RNA triggers antiviral signalling in humans. Nature (2018) 560(7717):238–42. doi: 10.1038/s41586-018-0363-0 PMC6570621 30046113

[25] Vedrenne V Gowher A De Lonlay P Nitschke P Serre V Boddaert N. Mutation in PNPT1, which encodes a polyribonucleotide nucleotidyltransferase, impairs RNA import into mitochondria and causes respiratory-chain deficiency. Am J Hum Genet (2012) 91(5):912–8. doi: 10.1016/j.ajhg.2012.09.001 PMC3487136 23084291

[26] Rice GI Bond J Asipu A Brunette RL Manfield IW Carr IM. Mutations involved in aicardi-goutières syndrome implicate SAMHD1 as regulator of the innate immune response. Nat Genet (2009) 41(7):829–32. doi: 10.1038/ng.373 PMC4154505 19525956

[27] Maharana S Kretschmer S Hunger S Yan X Kuster D Traikov S. SAMHD1 controls innate immunity by regulating condensation of immunogenic self RNA. Mol Cell (2022) 82(19):3712–28.e10. doi: 10.1016/j.molcel.2022.08.031 36150385

[28] Liddicoat BJ Piskol R Chalk AM Ramaswami G Higuchi M Hartner JC. RNA Editing by ADAR1 prevents MDA5 sensing of endogenous dsRNA as nonself. Science (2015) 349(6252):1115–20. doi: 10.1126/science.aac7049 PMC5444807 26275108

[29] Heraud-Farlow JE Walkley CR. The role of RNA editing by ADAR1 in prevention of innate immune sensing of self-RNA. J Mol Med (Berl). (2016) 94(10):1095–102. doi: 10.1007/s00109-016-1416-1 27044320

[30] Pestal K Funk Cory C Snyder Jessica M Price Nathan D Treuting Piper M Stetson Daniel B. Isoforms of RNA-editing enzyme ADAR1 independently control nucleic acid sensor MDA5-driven autoimmunity and multi-organ development. Immunity (2015) 43(5):933–44. doi: 10.1016/j.immuni.2015.11.001 PMC4654992 26588779

[31] Rice GI Kasher PR Forte GM Mannion NM Greenwood SM Szynkiewicz M. Mutations in ADAR1 cause aicardi-goutières syndrome associated with a type I interferon signature. Nat Genet (2012) 44(11):1243–8. doi: 10.1038/ng.2414 PMC4154508 23001123

[32] Li Q Gloudemans MJ Geisinger JM Fan B Aguet F Sun T. RNA Editing underlies genetic risk of common inflammatory diseases. Nature (2022) 608(7923):569–77. doi: 10.1038/s41586-022-05052-x PMC9790998 35922514

[33] Sun T Li Q Geisinger JM Hu S-B Fan B Su S. A small subset of cytosolic dsRNAs must be edited by ADAR1 to evade MDA5-mediated autoimmunity. Biorxiv (2022) 2022:08.29.505707. doi: 10.1101/2022.08.29.505707

[34] Peck LJ Wang JC. Energetics of b-to-Z transition in DNA. Proc Natl Acad Sci U S A. (1983) 80(20):6206–10. doi: 10.1073/pnas.80.20.6206 PMC394264 6578505

[35] Wang AH Quigley GJ Kolpak FJ Crawford JL van Boom JH van der Marel G. Molecular structure of a left-handed double helical DNA fragment at atomic resolution. Nature (1979) 282(5740):680–6. doi: 10.1038/282680a0 514347

[36] Herbert A. Z-DNA and z-RNA in human disease. Commun Biol (2019) 2:7. doi: 10.1038/s42003-018-0237-x 30729177 PMC6323056

[37] Nichols PJ Bevers S Henen M Kieft JS Vicens Q Vögeli B. Recognition of non-CpG repeats in alu and ribosomal RNAs by the z-RNA binding domain of ADAR1 induces a-z junctions. Nat Commun (2021) 12(1):793. doi: 10.1038/s41467-021-21039-0 33542240 PMC7862695

[38] Stellos K Gatsiou A Stamatelopoulos K Perisic Matic L John D Lunella FF. Adenosine-to-inosine RNA editing controls cathepsin s expression in atherosclerosis by enabling HuR-mediated post-transcriptional regulation. Nat Med (2016) 22(10):1140–50. doi: 10.1038/nm.4172 27595325

[39] Tang Q. Z-nucleic acids: uncovering the functions from past to present. Eur J Immunol (2022) 52:1700–11. doi: 10.1002/eji.202249968 PMC9827954 36165274

[40] Baranello L Wojtowicz D Cui K Devaiah BN Chung HJ Chan-Salis KY. RNA Polymerase II regulates topoisomerase 1 activity to favor efficient transcription. Cell (2016) 165(2):357–71. doi: 10.1016/j.cell.2016.02.036 PMC4826470 27058666

[41] Kouzine F Sanford S Elisha-Feil Z Levens D. The functional response of upstream DNA to dynamic supercoiling in vivo. Nat Struct Mol Biol (2008) 15(2):146–54. doi: 10.1038/nsmb.1372 18193062

[42] Liu H Mulholland N Fu H Zhao K. Cooperative activity of BRG1 and z-DNA formation in chromatin remodeling. Mol Cell Biol (2006) 26(7):2550–9. doi: 10.1128/MCB.26.7.2550-2559.2006 PMC1430323 16537901

[43] Herbert A. To "Z" or not to "Z": z-RNA, self-recognition, and the MDA5 helicase. PloS Genet (2021) 17(5):e1009513. doi: 10.1371/journal.pgen.1009513 33983939 PMC8118290

[44] de Reuver R Dierick E Wiernicki B Staes K Seys L De Meester E. ADAR1 interaction with z-RNA promotes editing of endogenous double-stranded RNA and prevents MDA5-dependent immune activation. Cell Rep (2021) 36(6):109500. doi: 10.1016/j.celrep.2021.109500 34380029

[45] Nakahama T Kato Y Shibuya T Inoue M Kim JI Vongpipatana T. Mutations in the adenosine deaminase ADAR1 that prevent endogenous z-RNA binding induce aicardi-goutières-syndrome-like encephalopathy. Immunity (2021) 54(9):1976–88.e7. doi: 10.1016/j.immuni.2021.08.022 34525338

[46] Tang Q Rigby RE Young GR Hvidt AK Davis T Tan TK. Adenosine-to-inosine editing of endogenous z-form RNA by the deaminase ADAR1 prevents spontaneous MAVS-dependent type I interferon responses. Immunity (2021) 54(9):1961–75.e5. doi: 10.1016/j.immuni.2021.08.011 34525337 PMC8459395

[47] Dias Junior AG Sampaio NG Rehwinkel J. A balancing act: MDA5 in antiviral immunity and autoinflammation. Trends Microbiol (2018) 27:75–85. doi: 10.1016/j.tim.2018.08.007 PMC6319154 30201512

[48] Chiang JJ Sparrer KMJ van Gent M Lässig C Huang T Osterrieder N. Viral unmasking of cellular 5S rRNA pseudogene transcripts induces RIG-i-mediated immunity. Nat Immunol (2018) 19(1):53–62. doi: 10.1038/s41590-017-0005-y 29180807 PMC5815369

[49] Zhao Y Ye X Dunker W Song Y Karijolich J. RIG-I like receptor sensing of host RNAs facilitates the cell-intrinsic immune response to KSHV infection. Nat Commun (2018) 9(1):4841. doi: 10.1038/s41467-018-07314-7 30451863 PMC6242832

[50] Hahne JC Lampis A Valeri N. Vault RNAs: hidden gems in RNA and protein regulation. Cell Mol Life Sci (2021) 78(4):1487–99. doi: 10.1007/s00018-020-03675-9 PMC7904556 33063126

[51] Vabret N Najburg V Solovyov A Gopal R McClain C Šulc P. Y RNAs are conserved endogenous RIG-I ligands across RNA virus infection and are targeted by HIV-1. iScience (2022) 25(7):104599. doi: 10.1016/j.isci.2022.104599 35789859 PMC9250025

[52] Kowalski MP Krude T. Functional roles of non-coding y RNAs. Int J Biochem Cell Biol (2015) 66:20–9. doi: 10.1016/j.biocel.2015.07.003 PMC4726728 26159929

[53] Schmidt N Domingues P Golebiowski F Patzina C Tatham MH Hay RT. An influenza virus-triggered SUMO switch orchestrates co-opted endogenous retroviruses to stimulate host antiviral immunity. Proc Natl Acad Sci USA (2019) 116(35):17399–408. doi: 10.1073/pnas.1907031116 PMC6717285 31391303

[54] Ji ZX Wang XQ Liu XF. NS1: a key protein in the "Game" between influenza a virus and host in innate immunity. Front Cell Infect Microbiol (2021) 11:670177. doi: 10.3389/fcimb.2021.670177 34327148 PMC8315046

[55] Ferreira CR Crow YJ Gahl WA Gardner PJ Goldbach-Mansky R Hur S. DDX58 and classic singleton-merten syndrome. J Clin Immunol (2019) 39(1):75–80. doi: 10.1007/s10875-018-0572-1 30574673 PMC6394545

[56] Jang MA Kim EK Now H Nguyen NT Kim WJ Yoo JY. Mutations in DDX58, which encodes RIG-I, cause atypical singleton-merten syndrome. Am J Hum Genet (2015) 96(2):266–74. doi: 10.1016/j.ajhg.2014.11.019 PMC4320253 25620203

[57] Prasov L Bohnsack BL El Husny AS Tsoi LC Guan B Kahlenberg JM. DDX58(RIG-i)-related disease is associated with tissue-specific interferon pathway activation. J Med Genet (2022) 59(3):294–304. doi: 10.1136/jmedgenet-2020-107447 33495304 PMC8310534

[58] Zheng J Wang C Chang MR Devarkar SC Schweibenz B Crynen GC. HDX-MS reveals dysregulated checkpoints that compromise discrimination against self RNA during RIG-I mediated autoimmunity. Nat Commun (2018) 9(1):5366. doi: 10.1038/s41467-018-07780-z 30560918 PMC6299088

[59] Lei Y Fei P Song B Shi W Luo C Luo D. A loosened gating mechanism of RIG-I leads to autoimmune disorders. Nucleic Acids Res (2022) 50(10):5850–63. doi: 10.1093/nar/gkac361 PMC9177982 35580046

[60] Li X Ranjith-Kumar CT Brooks MT Dharmaiah S Herr AB Kao C. The RIG-i-like receptor LGP2 recognizes the termini of double-stranded RNA. J Biol Chem (2009) 284(20):13881–91. doi: 10.1074/jbc.M900818200 PMC2679488 19278996

[61] Sanchez David RY Combredet C Najburg V Millot GA Beauclair G Schwikowski B. LGP2 binds to PACT to regulate RIG-I– and MDA5-mediated antiviral responses. Sci Signaling (2019) 12(601):eaar3993. doi: 10.1126/scisignal.aar3993 31575732

[62] Duic I Tadakuma H Harada Y Yamaue R Deguchi K Suzuki Y. Viral RNA recognition by LGP2 and MDA5, and activation of signaling through step-by-step conformational changes. Nucleic Acids Res (2020) 48(20):11664–74. doi: 10.1093/nar/gkaa935 PMC7672446 33137199

[63] Stok JE Oosenbrug T Ter Haar LR Gravekamp D Bromley CP Zelenay S. RNA Sensing via the RIG-i-like receptor LGP2 is essential for the induction of a type I IFN response in ADAR1 deficiency. EMBO J (2022) 41(6):e109760. doi: 10.15252/embj.2021109760 35156720 PMC8922249

[64] Takaoka A Wang Z Choi MK Yanai H Negishi H Ban T. DAI (DLM-1/ZBP1) is a cytosolic DNA sensor and an activator of innate immune response. Nature (2007) 448(7152):501–5. doi: 10.1038/nature06013 17618271

[65] Ha SC Kim D Hwang H-Y Rich A Kim Y-G Kim KK. The crystal structure of the second z-DNA binding domain of human DAI (ZBP1) in complex with z-DNA reveals an unusual binding mode to z-DNA. Proc Natl Acad Sci (2008) 105(52):20671–6. doi: 10.1073/pnas.0810463106 PMC2634953 19095800

[66] Kesavardhana S Kanneganti TD. ZBP1: a STARG ÅTE to decode the biology of z-nucleic acids in disease. J Exp Med (2020) 217(7):e20200885. doi: 10.1084/jem.20200885 32584411 PMC7336316

[67] Lin J Kumari S Kim C Van TM Wachsmuth L Polykratis A. RIPK1 counteracts ZBP1-mediated necroptosis to inhibit inflammation. Nature (2016) 540(7631):124–8. doi: 10.1038/nature20558 PMC5755685 27819681

[68] Maelfait J Liverpool L Bridgeman A Ragan KB Upton JW Rehwinkel J. Sensing of viral and endogenous RNA by ZBP1/DAI induces necroptosis. EMBO J (2017) 36(17):2529–43. doi: 10.15252/embj.201796476 PMC5579359 28716805

[69] Kuriakose T Man SM Malireddi RK Karki R Kesavardhana S Place DE. ZBP1/DAI is an innate sensor of influenza virus triggering the NLRP3 inflammasome and programmed cell death pathways. Sci Immunol (2016) 1(2):aag2045. doi: 10.1126/sciimmunol.aag2045 27917412 PMC5131924

[70] Zhang T Yin C Boyd DF Quarato G Ingram JP Shubina M. Influenza virus z-RNAs induce ZBP1-mediated necroptosis. Cell (2020) 180(6):1115–29.e13. doi: 10.1016/j.cell.2020.02.050 32200799 PMC7153753

[71] Wang Y Pandian N Han JH Sundaram B Lee S Karki R. Single cell analysis of PANoptosome cell death complexes through an expansion microscopy method. Cell Mol Life Sci (2022) 79(10):531. doi: 10.1007/s00018-022-04564-z 36169732 PMC9545391

[72] Jiao H Wachsmuth L Kumari S Schwarzer R Lin J Eren RO. Z-nucleic-acid sensing triggers ZBP1-dependent necroptosis and inflammation. Nature (2020) 580(7803):391 – 5. doi: 10.1038/s41586-020-2129-8 PMC7279955 32296175

[73] Devos M Tanghe G Gilbert B Dierick E Verheirstraeten M Nemegeer J. Sensing of endogenous nucleic acids by ZBP1 induces keratinocyte necroptosis and skin inflammation. J Exp Med (2020) 217(7):e20191913. doi: 10.1084/jem.20191913 32315377 PMC7336309

[74] Jiao H Wachsmuth L Wolf S Lohmann J Nagata M Kaya GG. ADAR1 averts fatal type I interferon induction by ZBP1. Nature (2022) 607(7920):776–83. doi: 10.1038/s41586-022-04878-9 PMC9329096 35859176

[75] Hubbard NW Ames JM Maurano M Chu LH Somfleth KY Gokhale NS. ADAR1 mutation causes ZBP1-dependent immunopathology. Nature (2022) 607(7920):769–75. doi: 10.1038/s41586-022-04896-7 PMC9339495 35859177

[76] Karki R Sundaram B Sharma BR Lee S Malireddi RKS Nguyen LN. ADAR1 restricts ZBP1-mediated immune response and PANoptosis to promote tumorigenesis. Cell Rep (2021) 37(3):109858. doi: 10.1016/j.celrep.2021.109858 34686350 PMC8853634

[77] Zhang T Yin C Fedorov A Qiao L Bao H Beknazarov N. ADAR1 masks the cancer immunotherapeutic promise of ZBP1-driven necroptosis. Nature (2022) 606(7914):594–602. doi: 10.1038/s41586-022-04753-7 35614224 PMC9373927

[78] Liu Y Cao H Zhao Y Shan L Lan S. Fisetin-induced cell death in human ovarian cancer cell lines via zbp1-mediated necroptosis. J Ovarian Res (2022) 15(1):57. doi: 10.1186/s13048-022-00984-4 35538559 PMC9092675

[79] Xu Z Williams BR. Genomic features of human PKR: alternative splicing and a polymorphic CGG repeat in the 5'-untranslated region. J Interferon Cytokine Res (1998) 18(8):609–16. doi: 10.1089/jir.1998.18.609 9726442

[80] Dar AC Dever TE Sicheri F. Higher-order substrate recognition of eIF2alpha by the RNA-dependent protein kinase PKR. Cell (2005) 122(6):887–900. doi: 10.1016/j.cell.2005.06.044 16179258

[81] Kim Y Lee JH Park JE Cho J Yi H Kim VN. PKR is activated by cellular dsRNAs during mitosis and acts as a mitotic regulator. Genes Dev (2014) 28(12):1310–22. doi: 10.1101/gad.242644.114 PMC4066401 24939934

[82] Harding HP Zhang Y Zeng H Novoa I Lu PD Calfon M. An integrated stress response regulates amino acid metabolism and resistance to oxidative stress. Mol Cell (2003) 11(3):619–33. doi: 10.1016/S1097-2765(03)00105-9 12667446

[83] Zamanian-Daryoush M Mogensen TH DiDonato JA Williams BR. NF-kappaB activation by double-stranded-RNA-activated protein kinase (PKR) is mediated through NF-kappaB-inducing kinase and IkappaB kinase. Mol Cell Biol (2000) 20(4):1278–90. doi: 10.1128/MCB.20.4.1278-1290.2000 PMC85265 10648614

[84] Chitrakar A Solorio-Kirpichyan K Prangley E Rath S Du J Korennykh A. Introns encode dsRNAs undetected by RIG-I/MDA5/interferons and sensed via RNase l. Proc Natl Acad Sci (2021) 118(46):e2102134118. doi: 10.1073/pnas.2102134118 34772806 PMC8609619

[85] Ishizuka JJ Manguso RT Cheruiyot CK Bi K Panda A Iracheta-Vellve A. Loss of ADAR1 in tumours overcomes resistance to immune checkpoint blockade. Nature (2018) 565:43–8. doi: 10.1038/s41586-018-0768-9 PMC7241251 30559380

[86] Maurano M Snyder JM Connelly C Henao-Mejia J Sidrauski C Stetson DB. Protein kinase r and the integrated stress response drive immunopathology caused by mutations in the RNA deaminase ADAR1. Immunity (2021) 54:1948–60. doi: 10.1101/2020.11.30.405498 PMC8446335 34343497

[87] Kim Y Park J Kim S Kim M Kang M-G Kwak C. PKR senses nuclear and mitochondrial signals by interacting with endogenous double-stranded RNAs. Mol Cell (2018) 71(6):1051–63.e6. doi: 10.1016/j.molcel.2018.07.029 30174290

[88] Kim S Lee K Choi YS Ku J Kim H Kharbash R. Mitochondrial double-stranded RNAs govern the stress response in chondrocytes to promote osteoarthritis development. Cell Rep (2022) 40(6):111178. doi: 10.1016/j.celrep.2022.111178 35947956

[89] Liu CX Li X Nan F Jiang S Gao X Guo SK. Structure and degradation of circular RNAs regulate PKR activation in innate immunity. Cell (2019) 177(4):865–80.e21. doi: 10.1016/j.cell.2019.03.046 31031002

[90] Farabaugh KT Krokowski D Guan BJ Gao Z Gao XH Wu J. PACT-mediated PKR activation acts as a hyperosmotic stress intensity sensor weakening osmoadaptation and enhancing inflammation. Elife (2020) 9:e52241. doi: 10.7554/eLife.52241 32175843 PMC7145421

[91] Ito T Yang M May WS. RAX, a cellular activator for double-stranded RNA-dependent protein kinase during stress signaling. J Biol Chem (1999) 274(22):15427–32. doi: 10.1074/jbc.274.22.15427 10336432

[92] Patel CV Handy I Goldsmith T Patel RC. PACT, a stress-modulated cellular activator of interferon-induced double-stranded RNA-activated protein kinase, PKR. J Biol Chem (2000) 275(48):37993–8. doi: 10.1074/jbc.M004762200 10988289

[93] Davidson S Yu CH Steiner A Ebstein F Baker PJ Jarur-Chamy V. Protein kinase r is an innate immune sensor of proteotoxic stress via accumulation of cytoplasmic IL-24. Sci Immunol (2022) 7(68):eabi6763. doi: 10.1126/sciimmunol.abi6763 35148201 PMC11036408

[94] Zhu J Zhang Y Ghosh A Cuevas Rolando A Forero A Dhar J. Antiviral activity of human OASL protein is mediated by enhancing signaling of the RIG-I RNA sensor. Immunity (2014) 40(6):936–48. doi: 10.1016/j.immuni.2014.05.007 PMC4101812 24931123

[95] Hartmann R Justesen J Sarkar SN Sen GC Yee VC. Crystal structure of the 2'-specific and double-stranded RNA-activated interferon-induced antiviral protein 2'-5'-oligoadenylate synthetase. Mol Cell (2003) 12(5):1173–85. doi: 10.1016/S1097-2765(03)00433-7 14636576

[96] Donovan J Dufner M Korennykh A. Structural basis for cytosolic double-stranded RNA surveillance by human oligoadenylate synthetase 1. Proc Natl Acad Sci USA (2013) 110(5):1652–7. doi: 10.1073/pnas.1218528110 PMC3562804 23319625

[97] Donovan J Whitney G Rath S Korennykh A. Structural mechanism of sensing long dsRNA via a noncatalytic domain in human oligoadenylate synthetase 3. Proc Natl Acad Sci USA (2015) 112(13):3949–54. doi: 10.1073/pnas.1419409112 PMC4386348 25775560

[98] Floyd-Smith G Slattery E Lengyel P. Interferon action: RNA cleavage pattern of a (2'-5')oligoadenylate–dependent endonuclease. Science (1981) 212(4498):1030–2. doi: 10.1126/science.6165080 6165080

[99] Donovan J Rath S Kolet-Mandrikov D Korennykh A. Rapid RNase l-driven arrest of protein synthesis in the dsRNA response without degradation of translation machinery. Rna (2017) 23(11):1660–71. doi: 10.1261/rna.062000.117 PMC5648034 28808124

[100] Siddiqui MA Mukherjee S Manivannan P Malathi K. RNase l cleavage products promote switch from autophagy to apoptosis by caspase-mediated cleavage of beclin-1. Int J Mol Sci (2015) 16(8):17611–36. doi: 10.3390/ijms160817611 PMC4581211 26263979

[101] Malathi K Dong B Gale M Silverman RH. Small self-RNA generated by RNase l amplifies antiviral innate immunity. Nature (2007) 448(7155):816–9. doi: 10.1038/nature06042 PMC3638316 17653195

[102] Koul A Deo S Booy EP Orriss GL Genung M McKenna SA. Impact of double-stranded RNA characteristics on the activation of human 2'-5'-oligoadenylate synthetase 2 (OAS2). Biochem Cell Biol (2020) 98(1):70–82. doi: 10.1139/bcb-2019-0060 30965010

[103] Wang Y Holleufer A Gad HH Hartmann R. Length dependent activation of OAS proteins by dsRNA. Cytokine (2020) 126:154867. doi: 10.1016/j.cyto.2019.154867 31629990

[104] Schwartz SL Park EN Vachon VK Danzy S Lowen AC Conn GL. Human OAS1 activation is highly dependent on both RNA sequence and context of activating RNA motifs. Nucleic Acids Res (2020) 48(13):7520–31. doi: 10.1093/nar/gkaa513 PMC7367156 32678884

[105] Banerjee S Gusho E Gaughan C Dong B Gu X Holvey-Bates E. OAS-RNase l innate immune pathway mediates the cytotoxicity of a DNA-demethylating drug. Proc Natl Acad Sci (2019) 116(11):201815071. doi: 10.1073/pnas.1815071116 PMC6421468 30814222

[106] Magg T Okano T Koenig LM Boehmer DFR Schwartz SL Inoue K. Heterozygous OAS1 gain-of-function variants cause an autoinflammatory immunodeficiency. Sci Immunol (2021) 6(60):eabf9564. doi: 10.1126/sciimmunol.abf9564 34145065 PMC8392508

[107] Manivannan P Reddy V Mukherjee S Clark KN Malathi K. RNase l induces expression of a novel Serine/Threonine protein kinase, DRAK1, to promote apoptosis. Int J Mol Sci (2019) 20(14):3535. doi: 10.3390/ijms20143535 31330998 PMC6679093

[108] Alagarasu K Honap T Damle IM Mulay AP Shah PS Cecilia D. Polymorphisms in the oligoadenylate synthetase gene cluster and its association with clinical outcomes of dengue virus infection. Infect Genet Evol (2013) 14:390–5. doi: 10.1016/j.meegid.2012.12.021 23337612

[109] Li Y Banerjee S Wang Y Goldstein SA Dong B Gaughan C. Activation of RNase l is dependent on OAS3 expression during infection with diverse human viruses. Proc Natl Acad Sci (2016) 113(8):2241–6. doi: 10.1073/pnas.1519657113 PMC4776461 26858407

[110] Thamizhmani R Vijayachari P. Association of dengue virus infection susceptibility with polymorphisms of 2'-5'-oligoadenylate synthetase genes: a case-control study. Braz J Infect Dis (2014) 18(5):548–50. doi: 10.1016/j.bjid.2014.03.004 PMC9428209 24819159

